# Longitudinal health survey of women from Venezuela in Colombia (ELSA-VENCOL): First report

**DOI:** 10.1371/journal.pone.0274157

**Published:** 2023-03-30

**Authors:** Jorge Acosta-Reyes, Julián Alfredo Fernández-Niño, Maylen Liseth Rojas-Botero, Laura Juliana Bonilla-Tinoco, Melissa Aguirre, Luis Ángel Anillo, David Alejandro Rodríguez, Lida Yoana Cifuentes, Iván Jiménez, Luisa Fernanda León, Ietza Bojorquez-Chapela

**Affiliations:** 1 Department of Public Health, Universidad del Norte, Barranquilla, Atlántico, Colombia; 2 Johns Hopkins Bloomberg School of Public Health, Johns Hopkins University, Baltimore, Maryland, United States of America; 3 Facultad Nacional de Salud Pública, Universidad de Antioquia, Medellín, Antioquia, Colombia; 4 Universidad Industrial de Santander, Bucaramanga, Santander, Colombia; 5 International Organization for Migration (IOM), Bogotá, Colombia; 6 El Colegio de la Frontera Norte, Tijuana, México; Fundación Universitaria del Área Andina, COLOMBIA

## Abstract

**Background:**

Colombia is currently the world’s main recipient country for Venezuelan migrants, and women represent a high proportion of them. This article presents the first report of a cohort of Venezuelan migrant women entering Colombia through Cúcuta and its metropolitan area. The study aimed to describe the health status and access to healthcare services among Venezuelan migrant women in Colombia with irregular migration status, and to analyze changes in those conditions at a one-month follow-up.

**Methods:**

We carried out a longitudinal cohort study of Venezuelan migrant women, 18 to 45 years, who entered Colombia with an irregular migration status. Study participants were recruited in Cúcuta and its metropolitan area. At baseline, we administered a structured questionnaire including sociodemographic characteristics, migration history, health history, access to health services, sexual and reproductive health, practice of early detection of cervical cancer and breast cancer, food insecurity, and depressive symptoms. The women were again contacted by phone one month later, between March and July 2021, and a second questionnaire was applied.

**Results:**

A total of 2,298 women were included in the baseline measurement and 56.4% could be contacted again at the one-month follow-up. At the baseline, 23.0% of the participants reported a self-perceived health problem or condition in the past month and 29.5% in the past 6 months, and 14.5% evaluated their health as fair or poor. A significant increase was found in the percentage of women who reported a self-perceived health problem during the past month (from 23.1% to 31.4%; p<0.01); as well as in the share who reported moderate, severe, or extreme difficulty working or performing daily chores (from 5.5% to 11.0%; p = 0.03) and who rated their health as fair (from 13.0% to 31.2%; p<0.01). Meanwhile, the percentage of women with depressive symptoms decreased from 80.5% to 71.2% (p<0.01).

**Conclusion:**

This report presents initial information on the health status of Venezuelan migrant women in Colombia, and is a starting point for further longer longitudinal follow-ups to assess changes over time in health conditions.

## Introduction

Since 2015, the social, political, and economic conditions in Venezuela have led to one of the largest international human mobility processes in the history of the Americas region. This process dramatically intensified in 2017, when the numbers of people irregularly entering Colombia (and other South American countries) from Venezuela increased significantly as a result of the exacerbation of the crisis especially after hyperinflation became the highest in the world [1, 2]. By November 2021, estimates showed roughly 6.04 million Venezuelan refugees and migrants worldwide [3]. This south-south migration [4] has involved a significant female participation, part of a global phenomenon known as “the feminization of the migration” [5].

With 1.84 million Venezuelan migrants in Colombia as of May 2022, the country is currently the world’s main recipient of these migrants in the world, most of them having entered the country irregularly [3]. In 2021, 81.3% of Venezuelan migrants in Colombia had an irregular migratory status or were still in the process of regularization [6]. In the same year, 49.0% of all Venezuelan migrants in Colombia were women [6].

Although Venezuelan migrants are a very socioeconomically diverse population, in the most recent migration waves many of them have been persons with high socioeconomic vulnerability [7, 8]. Thus, unlike the profile of migrants prior to 2017, the latest waves have been dominated by low- and middle-income irregular migrants who travel by land (in many cases by walking) to nearby countries, such as Colombia, which shares an 1,800 km border with Venezuela [1, 9].

Among the challenges faced by migrants are the health risks and needs associated with the entire migration process, including the conditions of departure, transit, and destination [10]. Since this is a mostly young population, the prevalence of chronic diseases is relatively low among them [9]. However, difficulties in accessing health services can cause them to get sick more frequently, which affects their well-being and could eventually impact health services in host countries [11]. In Colombia, it has been consistently reported that Venezuelan migrants are predominantly affected by skin, respiratory, gastrointestinal, immunopreventable, vector-borne, and infectious diseases (e.g., HIV/AIDS, tuberculosis, and sexually transmitted diseases), as well as by mental health disorders, gender-based violence, and health problems and needs related to pregnancy, childbirth, and the puerperium [11–15].

In addition, many migrants have encountered barriers to effectively access health services when they need them, even those services they are entitled to access according to current regulations in Colombia [11, 16]. This is due to, among other factors, having little knowledge about their rights, and to barriers in the access routes to health services [11, 17]. While Colombia’s health sector has had a response plan for migration [18] since 2018, most migrants with irregular immigration status can only access health services in emergencies or benefit from some public health interventions (such as vaccination), as well as receiving prenatal and natal care [18–20].

The Colombian government has made considerable efforts to expand the supply and coverage of health services to facilitate access to health services for Venezuelan migrants, as well as to keep records of the medical care that they receive [19]. Consequently, some of the existing information systems have been modified and new ones have been created for recording the medical care received by this population, as well as for vital statistics and epidemiological surveillance.

While national information systems have been important for monitoring the health needs of Venezuelan migrants and their use of health services, they still have several limitations [21]. Health information that is obtained from these sources largely depends on access to health services, which is low among migrants. Coverage is also limited and there are known lags and problems with the quality of migrants’ health information [21]. In addition, the scope of these data is limited, as they do not allow the use of a multidimensional approach to migrant health and are therefore insufficient to assess and monitor migrant health at the population level.

In addition, an integrated information system is still required to identify the situation and health needs of migrants, as well as their access to health services, especially for Venezuelan migrant women who represent approximately half of the migrant population. Migrant women are also the group of migrants that uses health services the most in Colombia, since the major number of outpatient consultations and hospitalizations for migrants in the past years in this country have been for pre-natal care and childbirth [22, 23]. Although the Department of National Statistics (DANE in Spanish) and several organizations have gone to great lengths to conduct population-based surveys, these tend to be limited to only a few dimensions and are mainly cross-sectional studies which are not able to evaluate changes in health status over time.

The generation of primary source information that includes individuals regardless of their having contact with health services can provide a better understanding of the health situation of Venezuelan migrant women in Colombia and other receiving countries in the Americas. In this descriptive study we aimed to identify the main health problems and the access to health services of irregular Venezuelan migrant women in Colombia, and the changes in both health problems and health care access with time after migration.

## Materials and methods

Requests for access to data could be sent to the Migration and Health Program of the International Organization for Migration in Colombia (iombogota@iom.int).

### Study design and population

This article reports on the first follow-up of a cohort of Venezuelan migrant women: ELSA-VENCOL (in Spanish: *Estudio longitudinal de Salud de las mujeres Venezolanas en Colombia*), one of the first longitudinal studies of Venezuelan migrants carried out in Colombia. The ELSA-VENCOL is a cohort study of Venezuelan migrant women with an irregular migration status who entered Colombia through Cúcuta and its metropolitan area, with a cross-sectional baseline measurement and a follow-up one month after. New follow-ups are expected to be done in the future.

**Fig 1:**
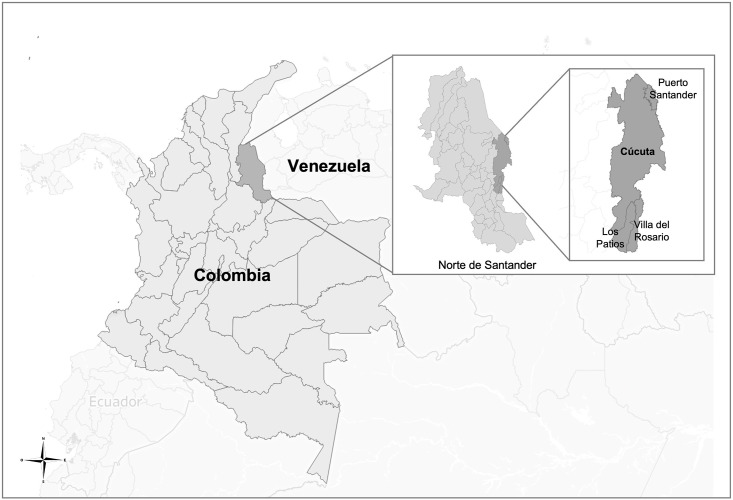
Location of Cúcuta and the three municipalities of its metropolitan area where the study population was recruited.

In Colombia, as in other Latin American countries, a “metropolitan area” is an urban region that includes a main city (the metropolis) that gives its name to the whole area and a series of cities or municipalities surrounding it, linked by territorial, environmental, economic, social, demographic, cultural and technological dynamics, and interrelationships. In this study, Cúcuta was included as the main city, alongside 3 of the 5 municipalities that constitute its metropolitan area: Villa del Rosario, Los Patios, and Puerto Santander. Fig 1 shows the place where the study was carried out, which has been historically the main land entry point for Venezuelan migrants to Colombia. During the last migrant wave, most entered the country walking through the regular and irregular points that exist in this area, before moving on to the larger cities or continuing to the south of the continent. This is also a region of high pendular flow (more than 35,000 people crossed per day before the pandemic), defined as the trans-border movement of people to obtain goods and services in one side of the border, coming back to their country of residence after just a few hours or days [3]. The flow in this region is thus mixed, including various types of migration. Although the flow decreased during the pandemic because of the closure that lasted until the end of 2021, the movement of people continued, mainly irregularly through informal crossings (called “*trochas*” in Spanish).

### Selection criteria, recruitment, and follow-up

This survey included women who: 1) were between 18 and 45 years of age; 2) had entered Colombia through Cúcuta (border city with Venezuela) or its metropolitan area (Los Patios, Puerto Santander or Villa del Rosario); 3) did not have Colombian nationality; 4) expressed the intention to remain in Colombia for at least one year; 5) were not “pendular” migrants (see definition below); and 6) did not have their passport stamped when entering Colombia and did not have a valid Special Permit to Stay (PEP in Spanish), which meant that their migration status was irregular.

For this study, “pendular” was defined as having entered Colombia two or more times during the month prior to the first interview for reasons other than tourism, vacations, family visits or business trips. That is, the study excluded persons who in the previous month had entered Colombia frequently for reasons of work, study or in search of services, among other purposes, since this population is part of the cross-border living dynamics rather than a migrant population.

The baseline interviews were conducted face-to-face between February 15 and May 25, 2021, at various locations in the cities of Cúcuta, Los Patios, Puerto Santander, and Villa del Rosario, and lasted an average of 30 minutes. In this interview, the telephone numbers of all participants were obtained, and they were informed that they would be contacted again one month later. For the follow-up interview, the women were reached by telephone between March 16 and July 6, 2021. The second contact was attempted beginning one month after the initial interview, at different times over a period of two weeks and on different days of the week. When there was no response after this period the participant was considered to be lost to follow-up. The average telephone call lasted 25 minutes.

Due to the mobile nature of the migrants, the interview took place in places of passage (bridges that cross the border, shelters, and other migrant concentration points), where the interviewer always sought a private space (tent, lounge) to conduct the interview. Participants did not receive any financial compensation for answering the survey since this was not logistically feasible.

### Sampling

Given the lack of a clearly defined sampling frame, a non-probability sampling method with snowball expansion was used. Interviewers went to temporary shelters and settlements where Venezuelan migrants were living and invited all migrants who were there during the fieldwork period to participate. Participants were also recruited on roads or bridges with high flows of migrants, and all Venezuelan women walking past that point were invited to participate.

### Data collection instruments and study variables

A standardized questionnaire with 161 questions was administered to assess the following dimensions of health status and access to health services: health history and perceived morbidity, effective access to health services, sexual and reproductive health, early detection of cervical and breast cancer, food insecurity and depressive symptoms.

The participants were asked about self-perceived health, self-perceived health problems or conditions over the past month, self-reported difficulty performing daily chores or work-related activities over the last six months (with a 6-item Likert scale ranging from none to extreme), medical diagnoses of diseases (presence or absence) and medication use (yes/no). These questions were extracted and adapted from the 2015 Colombia National Demographics and Health Survey (ENDS in Spanish). The presence of significant depressive symptoms was evaluated using the seven-item version of the Center for Epidemiological Studies depression scale (CES-D), with a cut-off point that was validated for the Colombian population (score greater than or equal to 8) [24–26].

Although the CES-D is not designed to establish the presence of a major depressive episode by itself, its use has been validated in population studies to detect "clinically significant depressive symptoms" [24, 27]. These symptoms may not always reflect clinical depression, yet they are still relevant from a public health perspective [28].

Sociodemographic information was also collected (age, marital status, whether the participant travelled alone or accompanied by others and number of people accompanying them, self-perceived ethnic group educational level, sleeping place, number of people in the household, head of household, income, subsidies received) as well as information about migration (country of residence, reason for migration, reason for entering Colombia through Cucuta, means of transportation, living in Colombia previously). The sociodemographic questions were adapted from the ENDS, and other questions were adapted from the 2018 National Population and Housing Census of Colombia and the 2007 Study on Global AGEing and Adult Health (SAGE) questionnaire.

All surveys were reviewed by experts on the topics and evaluated with a pilot test consisting of 30 interviews with migrants during the first two days of fieldwork (February 15 and 16, 2021), which allowed the questions to be adapted and modified. This pilot test demonstrated that the instruments and the items included performed well.

Similarly, a standardized questionnaire with 154 questions was used for the follow-up, which evaluated the same topics as those in the initial questionnaire, with the addition of questions on work history in Colombia. The follow-up questionnaire had fewer questions since those that were no longer relevant because it was not the first contact with the interviewers were eliminated. In addition, emphasis was placed on changes over the past month. Both interviews were conducted by previously trained interviewers.

### Statistical analysis

A descriptive analysis of the baseline data was performed using measures of central tendency and dispersion for quantitative variables and percentages for categorical variables. To assess changes between baseline and follow-up, bivariate analyses were performed with the McNemar test for dichotomous categorical variables and the marginal homogeneity test (Stuart-Maxwell) for nominal-polytomous variables. Ordinal variables were treated as polytomous categorical variables since we did not want to evaluate the correlation between the first and second measurements but rather the percentage change between the two measurements. For this reason, they were also analyzed using the marginal homogeneity test. In the case of quantitative variables, they were first evaluated using graphical and numerical methods (Shapiro-Wilk test) to identify whether they followed a normal distribution, and if they did not then the Wilcoxon test was used to compare the data from the two measurements.

To assess potential bias due to loss to follow-up, the baseline characteristics of the participants with and without follow-up were compared using the chi-square test for categorical variables (and Fisher’s exact test in cases where the sample was small) and the Mann-Whitney U test for quantitative variables.

All analyses were performed using Stata^®^ v14 with a significance level of 0.05 indicating statistically significant changes.

### Ethics statement

This study was reviewed and approved by the Research Ethics Committee of the Health Sciences Division from the Universidad del Norte (Acta: 202 of February 27, 2020).

All participants gave written informed consent to be included in the initial interview and their data were kept strictly confidential following the recommendations of the Ethics Committee. Each participant who signed the informed consent was accompanied by a witness, usually a person from the health sector or from a humanitarian mission who was not directly involved in this investigation. This witnesses, who was present during the entire first part of the interview, signed certifying that they oversaw the entire consent-taking process.

Also, telephone numbers were provided by the women after the study was explained and informed consent was obtained to use this numbers for a second interview via telephone. Informed oral consent was again obtained at the time of follow-up for each contacted participant following the same standardized procedures.

The interviewers were instructed to guarantee that people were fully aware that their participation in the survey was free and voluntary, without any prejudice and that it would not affect any social benefits that they may receive and that sharing the information would not pose any risk. To do this, they had to confirm that people understood the objective of the survey, their right to participate or not, and the guarantee of confidentiality. Even if women gave their written consent in the initial interview, they were free to refuse to participant in the follow-up, and this decision could be done or changed since the first contact, during the follow-up, or in any moment.

The capacity to consent was not explicitly assessed, however, each interviewer was trained to determine that the participant was fit to respond to the survey. All these procedures were approved by the ethics committee as part of the research protocol.

Personal information that could lead to individual identification of the participants was only known and handled by the research team during fieldwork and was omitted from the final databases. Specifically, the database was consolidated by a team from the International Organization for Migration (IOM), and all the observations were anonymized before being sent to the team from the Universidad del Norte that performed the statistical analysis without any access to the personal data of participants. The completed questionnaires were sent by the interviewers to the IOM team, who safeguarded them. No other team members, nor any external person, not even the sponsor had or has direct access to the original questionnaires with personal data.

## Results

A total of 2,298 Venezuelan migrant women were surveyed at baseline and 1,297 were surveyed at the one-month follow-up (56.4% response rate).

Statistically significant differences were found in the baseline measurements between women with and without follow-up. Specifically, those with follow-up had a higher educational level, had been in Colombia longer, and had more contact with Colombian health services. They also had a slightly smaller percentage of significant depressive symptoms (S1 Table).

### Socioeconomic and migration-related characteristics

In the baseline survey, the median age was 28 years (IQR: 23–35), 57.5% of the participants were married or in a free union, 59.4% were the head of household, 97.5% did not identify as belonging to any ethnic group and 50.4% had attained only secondary school education. In addition, 34.8% indicated that they would sleep on the street that night. The median number of household members in Colombia was 4 (IQR: 3–5), 72.4% of the households had only one income earner, 90.9% had a household monthly income of less than one minimum wage ($1,000,000 Colombian pesos per month, or approximately $259 USD) and only 0.9% received economic assistance or subsidies from the government (Table 1).

**Table 1:** Sociodemographic characteristics of the migrant women with irregular migration status participating in ELSA-VENCOL, 2021.

	Total baseline (n = 2298)	Sample with follow-up		p-value^c^
		Baseline (n = 1297)	Follow-up (n = 1297)	
	n (%)	n (%)	n (%)	
**Marital status**				<0.01
Married or free union	1322 (57.5)	799 (61.6)	678 (52.3)	
Single	518 (22.5)	272 (21.0)	563 (43.4)	
Separated, divorced, widowed	458 (19.9)	226 (17.4)	56 (4.3)	
**Ethnic group**				NE
Indigenous	15 (0.7)	5 (0.4)	NE	
Afro- descendant	33 (1.4)	17 (1.3)	NE	
Gypsy or Roma	9 (0.4)	4 (0.3)	NE	
None	2241 (97.5)	1271 (98.0)	NE	
**Educational level**				NE
None	24 (1.0)	12 (0.9)	NE	
Pre-school or elementary	929 (40.5)	491 (37.9)	NE	
Secondary education	1158 (50.4)	678 (52.3)	NE	
Higher education	187 (8.1)	116 (8.9)	NE	
**Where they spent the night**				<0.01
House or apartment of family members or someone known	121 (5.3)	81 (6.2)	170 (13.1)	
Rented house or apartment	725 (31.6)	529 (40.8)	517 (39.9)	
Rented room in house or apartment	119 (5.2)	78 (6.0)	286 (22.0)	
Tenement housing	233 (10.1)	151 (11.6)	131 (10.1)	
Hotel, hostel or guest house	1 (0.0)	1 (0.1)	1 (0.1)	
Shelter/migrant house	300 (13.1)	132 (10.2)	15 (1.2)	
Street	799 (34.8)	325 (25.1)	177 (13.6)	
**Number of migrant household members in Colombia** (median; IQR)	4 (3–5)	4 (3–5)	4 (3–5)	<0.01
Living alone	125 (5.4)	41 (3.2)	23 (1.8)	
2 to 4 people	1325 (57.7)	730 (56.3)	680 (52.4)	
5 or more people	848 (36.9)	526 (40.5)	59 (45.8)	
**Number of people contributing money to household expenses** (median; IQR)	1 (1–1)	1 (1–1)	1 (1–1)	0.55
None	75 (3.3)	41 (3.2)	80 (6.2)	
1	1665 (72.4)	966 (74.5)	899 (69.3)	
2+	558 (24.3)	290 (22.3)	318 (24.5)	
**Number of people who depend on that income**^a^ (median; IQR)	4 (3–5)	4 (3–5)	5 (4–6)	<0.01
1–2	294 (13.2)	134 (10.7)	61 (5.0)	
3–4	1016 (45.7)	578 (46.0)	511 (42.0)	
5 or more	913 (41.1)	544 (43.3)	645 (53.0)	
**Household income** ^a^^**,**^ ^b^				<0.01
Less than 1 SMMLV	2020 (90.9)	1146 (91.2)	1195 (98.2)	
Between 1 and < 3 SMMLV	8 (0.4)	6 (0.5)	22 (1.8)	
**Receiving financial help or subsidies**				<0.01
Yes	20 (0.9)	10 (0.8)	74 (5.7)	

^a^ These questions were only evaluated for migrants who indicated that at least one person was working and contributing money to household expenses.

^b^ Only the categories with responses were included

Current Minimum Monthly Legal Salary [SMMLV in Spanish] when the questionnaire was administered was $908.526

^c^. Hypothesis tests were conducted to compare the change between each of the variables in the baseline and the value at the follow-up at one month, only among those subjects who had a follow-up. The McNemar test was used for dichotomous categorical variables, the marginal homogeneity test (Stuart-Maxwell) for nominal-polytomous or ordinal variables, and the Shapiro-Wilk test for quantitative variables. NE = Not evaluated in the follow-up.

A decrease between baseline and follow-up was found in the percentage of migrants sleeping on the street, while an increase was found in the percentage of those who contributed money to the household and in participants whose households received economic assistance (Table 1). At follow-up, only 16.3% (n = 212) of the participants had initiated the process to regularize their migration status. The documents that were most frequently obtained were the PPT (n = 143; 67.5%) the PEP (34; 16.0%) and the laissez-passer (n = 35; 16.5%).

### Health status and access to health services

At baseline, 23.0% of the participants reported a self-perceived health problem or condition in the past month and 29.5% in the past 6 months, 13.1% evaluated their health as fair, and 1.1% as poor or very poor. The most frequent medical diagnosis was vector-borne disease (18.7%) and 82.0% of the migrants had significant depressive symptoms according to CES-D at the time of the interview. Of the participants who needed medications at the time of the interview, 36.4% were not taking them, primarily because of their cost (Table 2).

**Table 2:** Health status, access to healthcare services and self-perceived health among the migrant women with an irregular migration status participating in ELSA-VENCOL, 2021.

	Total baseline (n = 2298)	Sample with follow-up^a^		p-value^c^
		Baseline (n = 1297)	Follow-up (n = 1297)	
	n (%)	n (%)	n (%)	
**Some type of self-perceived health problem or condition**				
Over the past month	529 (23.0)	299 (23.1)	407 (31.4)	<0.01
Over the past 6 months	678 (29.5)	385 (29.7)	N/A	
**Difficulty performing daily chores or working**				0.03
None	1935 (84.2)	1083 (83.6)	930 (71.7)	
Mild	237 (10.3)	141 (10.9)	225 (17.3)	
Moderate/Severe/Extreme	125 (5.4)	72 (5.5)	142 (11.0)	
**Medical diagnosis** ^a^				
High blood pressure *	102 (4.4)	67 (5.2)	108 (4.7)	0.60
Diabetes	22 (1.0)	11 (0.9)	23 (1.8)	0.99
Dyslipidemia	24 (1.0)	14 (1.1)	27 (1.2)	0.25
HIV/AIDS	2 (0.1)	1 (0.1)	2 (0.1)	0.48
Tuberculosis	4 (0.2)	2 (0.2)	4 (0.3)	0.56
Hepatitis B or C	1 (0.0)	2 (0.0)	1 (0.0)	0.32
Vector-borne disease	430 (18.7)	255 (19.7)	430 (33.2)	<0.01
Depression	26 (1.1)	17 (1.3)	32 (1.4)	0.34
Other illnesses *	411 (17.9)	239 (18.4)	429 (18.7)	0.38
**Need to take medication**				
Yes	401 (17.5)	237 (18.3)	251 (19.4)	0.18
**Current use of the medication that is needed** *				
No	146 (36.4)	87 (36.7)	67 (26.7)	0.01
**Reason for not using needed medication**				0.31
It is very expensive or no way to pay for it	108 (74.0)	63 (72.4)	53 (79.1)	
Do not like the medications	9 (6.2)	6 (6.9)	1 (1.5)	
There was no medication when sought/it was not found	6 (4.1)	3 (3.5)	0 (0.0)	
Don’t know where to get it	5 (3.4)	2 (2.3)	2 (3.0)	
Others	18 (12.3)	13 (14.9)	11 (16.4)	
**Self-perceived health**				<0.01
Very good/good	1963 (85.5)	1110 (85.7)	875 (67.5)	
Fair	307 (13.4)	169 (13.0)	404 (31.1)	
Very poor/poor	26 (1.1)	17 (1.3)	18 (1.4)	
**Clinically significant depressive symptoms (CES-D)**				
Yes	1880 (82.0)	1043 (80.5)	923 (71.2)	<0.01

^a^ The follow-up sample column shows the global prevalence of the illness during the study period in the subsample of women who had a one-month follow-up, that is, the total people for whom the condition was evaluated over the course of the study was counted and divided by 1,297.

^c^. Hypothesis tests were conducted to compare the change between each of the variables in the baseline and the value at the follow-up at one month, only among those subjects who had a follow-up. The McNemar test was used for dichotomous categorical variables, the marginal homogeneity test (Stuart-Maxwell) for nominal-polytomous or ordinal variables, and the Shapiro-Wilk test for quantitative variables

In the sub-sample with follow-up data, we found significant changes after one month in some conditions. The self-perception of a health problem or condition in the last month increased by 35.9%, the proportion of women who reported severe difficulties in performing daily chores or working doubled, the use of necessary medications decreased by 27.2%, and the self-perception of their own health worsened, such that the percentage of those who considered their health was very good or good decreased by 21.2%, and the percentage of those who considered their health was fair increased by 139.2% (Table 2). The prevalences at the baselines and the changes in other health problems are also presented in Table 2.

The above findings were consistent when we analyzed subgroups according to their length of stay of in Colombia (Table 3), however, it is worth noting that those who had been less than a month in the country at the time of recruiting tended to have more pronounced changes. Thus, while those who had been in the country for more than a year presented an increase of 30.0% and 87.5% in the self-perception of a health problem and in the difficulties to carry out daily chores, respectively, the correspondent increase was of the order of 95, 1% and 197.0% for those who had been in the country for less than a month.

**Table 3:** Health characteristics, self-perceived morbidity and food security by length of stay in Colombia, among the migrant women with irregular migratory status participating in ELSA-VENCOL, 2021.

	Length of stay in Colombia											
	≤ 1 Month				> 1 month to 1 year				> 1 year			
	Total baseline657 (%)	Baseline with follow-up 184 (%)	Follow-up 184 (%)	p-value^a^	Total baseline 464 (%)	Baseline with follow-up 256 (%)	Follow-up 256 (%)	p-value^a^	Total Baseline 1177 (%)	Baseline with follow-up 857 (%)	Follow-up 857 (%)	p-value^a^
**Some type of self-perceived health problem or condition**												
Over the past month	123 (18.8)	26 (14.2)	51 (27.7)	< 0.01	128 (27.6)	70 (27.3)	92 (35.9)	0.026	278 (23.6)	203 (23.7)	264 (30.8)	< 0.01
Over the past 6 months	173 (26.4)	44 (24.0)	NA		140 (30.2)	71 (27.7)	NA		365 (31.0)	270 (31.5)		NA
**Difficulty performing chores or working**												
None	578 (88.1)	163 (88.6)	139 (75.5)	< 0.01	383 (82.5)	212 (82.8)	174 (68.0)	< 0.01	974 (82.8)	708 (82.6)	617 (72.0)	< 0.01
Slight	52 (7.9)	15 (8.2)	27 (14.7)		53 (11.4)	26 (10.2)	48 (18.8)		132 (11.2)	101 (11.8)	150 (17.5)	
Moderate/Severe/Extreme	26 (4.0)	6 (3.3)	18 (9.8)		28 (6.0)	18 (7.0)	34 (13.3)		71 (6.0)	48 (5.6)	90 (10.5)	
**Medical diagnosis**												
High blood pressure	26 (4.0)	11 (6.0)	11 (6.0)	NS	15 (3.2)	9 (3.5)	10 (3.9)	NS	61 (5.2)	47 (5.5)	52 (6.1)	0.063
Diabetes	7 (1.1)	1 (0.5)	1 (0.5)	NS	3 (0.6)	1 (0.4)	1 (0.4)	NS	12 (1.0)	9 (1.1)	10 (1.2)	NS
Dyslipidemia	9 (1.4)	2 (1.1)	4 (2.2)	NS	4 (0.9)	4 (1.6)	4 (1.6)	NS	11 (0.9)	8 (0.9)	9 (1.1)	NS
HIV/AIDS	0 (0.0)	0 (0.0)	0 (0.0)	NS	0 (0.0)	0 (0.0)	0 (0.0)	NS	2 (0.2)	1 (0.1)	1 (0.1)	NS
Tuberculosis	2 (0.3)	1 (0.5)	1 (0.5)	NS	0 (0.0)	0 (0.0)	0 (0.0)	NS	2 (0.2)	1 (0.1)	1 (0.1)	NS
Hepatitis B or C	0 (0.0)	0 (0.0)	0 (0.0)	NS	0 (0.0)	0 (0.0)	0 (0.0)	NS	1 (0.1)	0 (0.0)	0 (0.0)	NS
Vector-borne disease	133 (20.3)	33 (17.9)	33 (17.9)	NS	80 (17.2)	52 (20.3)	52 (20.3)	NS	217 (18.4)	170 (19.8)	170 (19.8)	NS
Depression	2 (0.3)	0 (0.0)	2 (1.1)	NS	7 (1.5)	3 (1.2)	5 (2.0)	NS	17 (1.4)	14 (1.6)	16 (1.9)	NS
Other illnesses	101 (15.4)	25 (13.6)	27 (14.7)	0.48	97 (20.9)	56 (21.9)	60 (23.4)	0.125	213 (18.1)	158 (18.4)	170 (19.8)	< 0.01
**Need to take medication**												
Yes	102 (15.6)	29 (15.8)	29 (15.8)	NS	86 (18.5)	51 (19.9)	56 (21.9)	0.405	213 (18.1)	157 (18.3)	166 (19.4)	0.298
**Taking the medication that is needed**												
No	45 (44.1)	9 (31.0)	5 (17.2)	0.22	30 (34.9)	18 (35.3)	13 (23.2)	0.169	71 (33.3)	60 (38.2)	49 (29.5)	0.098
**Reason for not taking the medication needed**												
It is very expensive or no way to pay for it	36 (80.0)	6 (66.7)	5 (100.0)	NS	20 (66.7)	13 (72.2)	10 (76.9)	NS	52 (73.2)	44 (73.3)	38 (77.6)	0.612
**Self-perceived health**												
Very good	189 (28.9)	48 (26.2)	11 (6.0)	< 0.01	157 (33.8)	86 (33.6)	31 (12.1)	< 0.01	455 (38.7)	339 (39.6)	132 (15.4)	< 0.01
Good	363 (55.4)	109 (59.6)	116 (63.0)		235 (50.6)	131 (51.2)	123 (48.0)		564 (47.9)	397 (46.3)	462 (53.9)	
Fair or poor	103 (15.7)	26 (14.2)	57 (31.0)		72 (15.5)	39 (15.2)	102 (39.8)		158 (13.4)	121 (14.1)	263 (30.7)	
**Depressive symptoms (CES-D)**												
Yes	550 (83.7)	152 (82.6)	138 (75.0)	0.082	383 (82.5)	203 (79.3)	175 (68.6)	< 0.01	947 (80.5)	688 (80.3)	610 (71.2)	< 0.01

* p value (McNemar Test) for the significance of the change in the prevalence between baseline and the follow-up (among women with follow-up). Hypothesis tests were conducted to compare the change between each of the variables in the baseline and the value at the follow-up at one month, only among those subjects who had a follow-up. The McNemar test was used for dichotomous categorical variables, the marginal homogeneity test (Stuart-Maxwell) for nominal-polytomous or ordinal variables.

NS: Non-significant

## Discussion

The present article reports on the health status and access to health services of Venezuelan migrant women with irregular migration status at baseline and one-month follow-up in ELSA-VENCOL, and is one of the first longitudinal studies of Venezuelan migrants carried out in Colombia.

The baseline information showed that roughly one-fifth of the participants had had a self-perceived health problem during the previous month. However, the prevalence of a prior diagnosis of chronic diseases was low. This might be due to the relatively young age of the women surveyed. A “healthy migrant” phenomenon, might also be in place in this population, as has been reported all over the world and in Latin America [29]. Alternatively, since the questionnaire asked about previous diagnosis of the chronic diseases, lack of access to health care could also play a part. At the same time, the prevalence of depressive symptoms (over the CES-D cutoff) was extremely high (82% at baseline), which likely reflects the psychosocial impact of the conditions associated with migration [30].

Over the follow-up period, the prevalence of significant depressive symptoms decreased. When also considering the improvement in socioeconomic factors (a higher percentage who had stable living conditions such as rented houses or rooms, received subsidies or had contact with health services), these results seem to indicate that more time in Colombia is associated with a more stable situation, which may explain the improved emotional state. Similarly, other authors have described [31–33] that the prevalence of mental health problems decreases after resettlement in the recipient country.

Moreover, the increase in the prevalence of reported health problems is an interesting finding of this study. To our knowledge, there are few studies of the changes in health profiles and health care utilization of recent migrants over a period similar to the one we report, but a study of Syrian refugees showed that after one year of resettlement in Norway they more than doubled their baseline use of general practitioners, which could reflect better access to care, and not only worsening health [31]. On the other hand, a cohort study of Bosnian refugees in Croatia found that, at a three-year follow-up, their health status had improved [34]. Health improvement would be expected since migrants tend to experience less socioeconomic risk factors for health problems (e.g. poverty, insecure housing) over time. However, it is possible that the time frame of our study did not capture this [35]. Instead, having had more access to services at follow up could make women more likely to perceive when they had a health problem, rather than dismiss it out of hand when unable to access treatment. In addition, the slight increase in the prevalence of some of the diseases evaluated could correspond to an increase in the detection of the pathology (possibly due to greater access to health services) rather than to a real increase in the disease. While given the data in this study these interpretations are speculative, it would be interesting to explore them further in the future.

There are two areas of the current discussion on the relationships of migration and health that our data can address. First, the literature in this field often employs the concept of the “healthy migrant phenomenon”, in which people who are healthier are more likely to migrate and therefore immigrants seem to have a health advantage in comparison with local populations [36]. However, this effect may not apply to all types of migrants, with forced migrants less likely to present favorable health profiles in comparison with the local non-migrant population, especially in regard to mental health. Since Venezuelan migrants are a mixed flow that shares many characteristics with forced migrants, studying this groups reminds us of how the association between human mobility and health depends on the causes and context of mobility. Secondly, while this study of a south-south migration replicates what has been shown for south-north migration, in the sense that settlement can be beneficial for health and access to health care, the fact that only 16.3% of the participants who were followed had sought to regularize their migration status, despite the policy of the Colombian government of promoting regularization [37], hints to barriers that may be particular of this context.

This study presents broad findings about health status among Venezuelan women migrants in Colombia, but its conclusions are certainly limited. The follow-up of only one month is too short to assess changes in various health outcomes, however we know that some acute health problems as well as mental health episodes could change rapidly, and we wanted to explore the changes as part of the initial phase of the analysis, also considering that in a longer period the loss to follow-up would be greater. However, this is certainly a limitation of the study, and we hope to be able to continue with longer-term follow-ups to assess changes over longer periods that could provide more valuable information. Another limitation of this study is the fact that the loss to follow-up was differential according to time in Colombia. Follow-up information was obtained from only 28% of those who had been in the country for one month or less at the time of the baseline survey, compared to 55% of those who had been in Colombia for more than one month and up to one year and 74% of those who had been in Colombia for more than one year. Thus, the conclusions obtained may not be fully generalizable to newly arrived migrants and those who are still in transit. However, the differences were not statistically significant for most of the variables. A further limitation is that, due to its very nature, this survey is not exempt from the possibility of information bias considering, for example, that the participants could face memory bias, or in other cases, interest in denouncing greater health needs waiting for an institutional response, despite the fact that the researchers tried not to generate expectations derived from the study.

Furthermore, the response rate was 56% at follow-up. This limitation needs to be considered when making practical decisions based on the data collected. In addition, there is the possibility of information bias because it was detected that some migrants gave erroneous answers thinking that they would receive a benefit. This could not be corrected for participants without follow-up. On the other hand, the strengths of this study include 1) its longitudinal design, 2) the population of women given their specific health needs and that they currently represent 49% of the Venezuelan migrant population in Colombia [5], and 3) the sample size, which provided sufficient statistical power for analyzing the data. This type of analysis could help to adapt interventions so that they are more specific to the target population group.

## Conclusion

This study was able to collect a large amount of information. It is hoped that this data will be used to generate research hypotheses for subsequent studies to investigate particular relationships or associations between variables, or to provide explanations for some of the findings. It is also hoped that this study will help to guide the design of public policies aimed at facilitating the inclusion of Venezuelan migrants into Colombian society and improving their quality of life, and it will also serve as a first step towards closing the knowledge gap with respect to social determinants, health status and access to health care in this population.

## Supporting information

S1 Table Comparison between participants with and without follow-up of the information from the baseline questionnaire administered to the migrant women with irregular migration status participating in ELSA-VENCOL, 2021. (DOCX)
