# Supplementary material for: Longitudinal health survey of women from Venezuela in Colombia (ELSA-VENCOL): First report
Source: PLoS One. 2023 Mar 30;18(3):e0274157. doi: 10.1371/journal.pone.0274157 (PMC10062563; doi:10.1371/journal.pone.0274157)
Supplement: S1 Table — (DOCX) [file pone.0274157.s001.docx]

**Table S1.** Comparison between participants with and without follow-up of the information from the baseline questionnaire administered to the migrant women with irregular migration status participating in ELSA-VENCOL, 2021

|  | **Follow-up** | | **p value^b^** |
| --- | --- | --- | --- |
|  | **No**  **[n= 1001]**  **n [%]** | **Yes**  **[n= 1297]**  **n [%]** |  |
| **SOCIODEMOGRAPHIC DATA** |  |  |  |
| **Age [median; IQR]** | 28  [22-35] | 29 [24-36] | 0.08 |
| **Marital status** |  |  | **<0.01** |
| Married or free union | 523 [52.2] | 799 [61.6] |  |
| Single | 246 [24.6] | 272 [21.0] |  |
| Separated, divorced or widowed | 232 [23.2] | 226 [17.4] |  |
| **Educational level** |  |  | **0.04** |
| None | 12 [1.2] | 12 [0.9] |  |
| Pre-school or elementary | 436 [43.8] | 489 [37.8] |  |
| Secondary school | 480 [47.9] | 678 [52.3] |  |
| Higher education | 71 [7.1] | 116 [8.9] |  |
| **Where the night was spent** |  |  | <0.01 |
| House or apartment of family members or someone known | 40 [4.0] | 81 [6.2] |  |
| Rented house or apartment | 196 [19.6] | 529 [40.8] |  |
| Rented room in house or apartment | 41 [4.1] | 78 [6.0] |  |
| Tenement housing | 82 [8.2] | 151 [11.6] |  |
| Hotel, hostel or guest house | 0 [0.0] | 1 [0.1] |  |
| Shelter/migrant house | 168 [16.8] | 132 [10.2] |  |
| Other [street, carp, tent, wagon, boat, natural shelter, bridge, park, etc.] | 325 [25.1] | 799 [34.8] |  |
| **Head of household** |  |  | 0.15 |
| Yes | 615 [61.4] | 751 [57.9] |  |
| **Number of household members in Colombia** [median; IQR] | 4 [3-5] | 4 [3-5] | <0.01 |
| **Number of people contributing money to household expenses** [median; IQR] | 1 [1-2] | 1 [1-1] | 0.02 |
| **Number of people who depend on that income^a^**  [median; IQR] | 4 [3-5] | 4 [3-5] | <0.01 |
| **Household income ^a, b^** |  |  | 0.38 |
| Less than 1 SMMLV | 874 [90.4] | 1146 [91.2] |  |
| Between 1 and < 3 SMMLV | 2 [0.2] | 6 [0.5] |  |
| Does not know/No response | 91 [9.4] | 104 [8.3] |  |
| **Receiving financial help or subsidies** |  |  | 0.75 |
| Yes | 10 [1.0] | 10 [0.8] |  |
| **MIGRATORY FLOW** |  |  |  |
| **Amount of time in Colombia ^b^** |  |  | <0.01 |
| 1 month or less | 473 [47.2] | 184 [14.2] |  |
| 1-6 months | 166 [16.6] | 179 [13.8] |  |
| >6 months – 1 year | 42 [4.2] | 77 [5.9] |  |
| More than 1 year | 320 [32.0] | 857 [66.1] |  |
| **HEALTH HISTORY AN SELF-PERCEIVED MORBIDITY** |  |  |  |
| **Some type of health problem or condition** |  |  |  |
| Over the past month | 230 [23.0] | 299 [23.1] | 0.96 |
| Over the past 6 months | 293 [29.3] | 385 [29.7] | 0.83 |
| **Difficulty doing chores or working** |  |  | 0.32 |
| None | 852 [85.1] | 1083 [83.6] |  |
| Mild | 96 [9.6] | 141 [10.9] |  |
| Moderate/Severe/Extreme | 53 [5.3] | 72 [5.5] |  |
| **Self-perceived health** |  |  | 0.18 |
| Very good | 328 [32.8] | 473 [36.5] |  |
| Good | 525 [52.5] | 637 [49.2] |  |
| Fair | 138 [13.8] | 169 [13.0] |  |
| Poor | 7 [0.7] | 16 [1.2] |  |
| Very poor | 2 [0.2] | 1 [0.1] |  |
| **SIGNIFICANT DEPRESSIVE SYMPTOMS [CES-D]** |  |  | 0.04 |
| Yes | 837 [83.9] | 1043 [80.5] |  |
| **ACCESS TO HEALTH SERVICES** |  |  |  |
| **Contact with the SSC** |  |  | **<0.01** |
| Yes | 336 [33.6] | 731 [56.4] |  |
| **Continuation of treatment after the medical visit ^a^** |  |  | 0.85 |
| Yes | 138 [41.1] | 302 [41.3] |  |
| No | 10 [3.0] | 27 [3.7] |  |
| No treatment required | 188 [55.9] | 402 [55.0] |  |
| **Participation in vaccination campaigns ^a^** |  |  | 0.10 |
| Yes | 121 [36.0] | 302 [41.3] |  |
| **Participation in health brigades ^a^** |  |  | <0.01 |
| Yes | 136 [40.5] | 374 [51.2] |  |
| **Hospitalization in Colombia ^a^** |  |  | 0.49 |
| Yes | 44 [13.1] | 85 [11.6] |  |

^a^ Questions asked only of those who indicated having had contact with Colombian health services [SSC in Spanish]

b. Hypothesis tests were conducted to compare the change between each of the variables in the baseline and the value at the follow-up at one month, only among those subjects who had a follow-up. The McNemar test was used for dichotomous categorical variables, the marginal homogeneity test (Stuart-Maxwell) for nominal-polytomous or ordinal variables, and the Shapiro-Wilk test for quantitative variables
